# Characterizing Grover search algorithm on large-scale superconducting quantum computers

**DOI:** 10.1038/s41598-024-80188-6

**Published:** 2025-01-08

**Authors:** Muhammad AbuGhanem

**Affiliations:** Faculty of Science, Ain Shams University, Cairo, 11566 Egypt

**Keywords:** Quantum search, Grover search algorithm, Quantum algorithms in real-world applications, Applied quantum computing, Quantum hardware characterization, State-of-the-art superconducting quantum computers, Quantum information, Computer science, Qubits

## Abstract

Quantum computing is on the cusp of transforming the way we tackle complex problems, and the Grover search algorithm exemplifying its potential to revolutionize the search for unstructured large datasets, offering remarkable speedups over classical methods. Here, we report results for the implementation and characterization of a three-qubit Grover search algorithm using the state-of-the-art scalable quantum computing technology of superconducting quantum architectures. To delve into the algorithm’s scalability and performance metrics, our investigation spans the execution of the algorithm across all eight conceivable single-result oracles, alongside nine two-result oracles, employing IBM Quantum’s 127-qubit quantum computers. Moreover, we conduct five quantum state tomography experiments to precisely gauge the behavior and efficiency of our implemented algorithm under diverse conditions – ranging from noisy, noise-free environments to the complexities of real-world quantum hardware. By connecting theoretical concepts with real-world experiments, this study not only shed light on the potential of Noisy Intermediate-Scale Quantum Computers in facilitating large-scale database searches but also offer valuable insights into the practical application of the Grover search algorithm in real-world quantum computing applications.

## Introduction

Quantum computing has emerged as a transformative field with the potential to revolutionize various domains, including quantum machine learning (QML)^1^, cryptography^2^, combinatorial optimization^3^, quantum chemistry^4^, drug discovery^5^, and long distance quantum communications^6,7^. At the heart of quantum computing lies the promise of harnessing quantum phenomena to perform computations at unprecedented speeds, surpassing the capabilities of classical computers^8–16^.

**Fig. 1:**
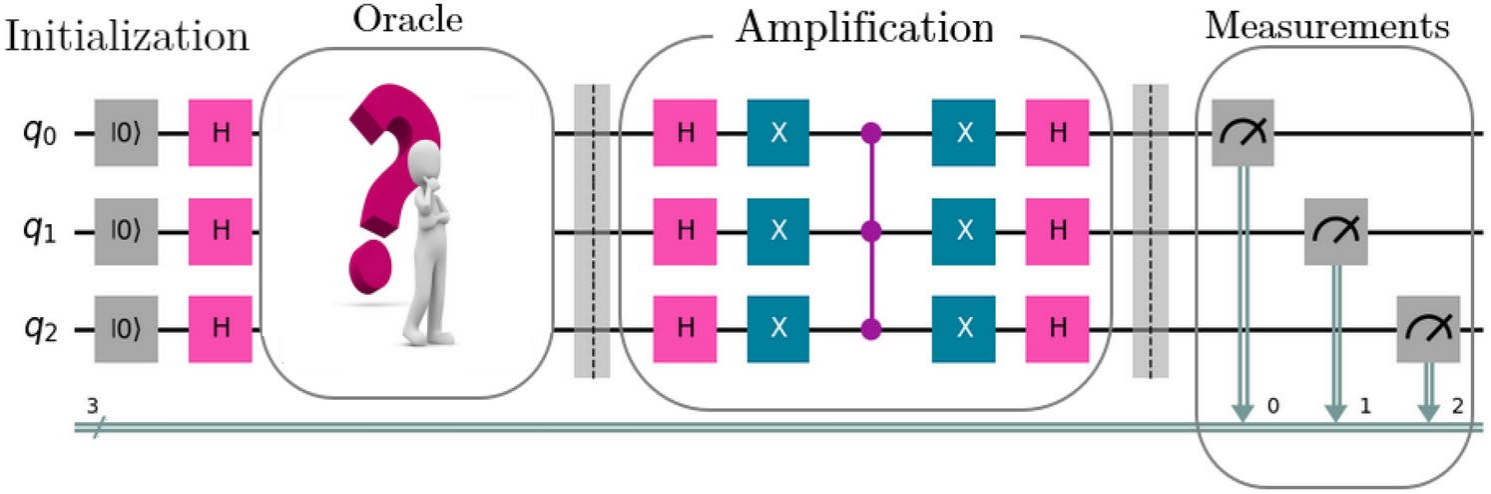
A schematic circuit representation illustrating the key stages of GSA. The process includes initialization, where the qubits are prepared in a superposition state; marking, where the target item (items) in the database are identified and marked by the oracle; amplification, where the amplitude(s) of the marked item(s) are increased; and measurement, where the final result is obtained. Here, $\left| 0\right\rangle$, H, and X represent the initialization of the qubit to the $\left| 0\right\rangle$ state, the Hadamard gate, and Pauli’s X gate, respectively.

Searching through extensive databases is a crucial challenge with far-reaching applications. Among the plethora of quantum algorithms developed thus far, Grover’s search algorithm (GSA) stands out as a powerful technique for searching unsorted databases^17,18^. Proposed by Lov Grover in 1996, this algorithm offers a quadratic speedup over classical algorithms, making it particularly attractive for a wide range of applications^17^.

Grover’s algorithm occupies a central position within the realm of quantum computing, heralded for its versatility and utility across numerous disciplines. It stands as the optimal search algorithm for quantum architectures^19^, finding utility as a foundational component for quantum algorithms^20,21^. Its applicability extends to facilitating string matching tasks^22^, addressing minimum search challenges^23^, tackling computational geometry problems^24^. and enabling quantum dynamic programming^25^. Successful demonstrations of searches employing two qubits have been achieved across different quantum platforms^26–32^. However, efforts to expand these capabilities to larger search spaces have primarily been demonstrated on non-scalable NMR (nuclear magnetic resonance) systems^33^. Additionally, a three-qubit GSA using programmable trapped atomic ion systems was implemented in^34^.

Recent advancements in implementing GSA on Noisy Intermediate-Scale Quantum (NISQ) devices and superconducting quantum platforms have significantly progressed. Notably, prior implementations have demonstrated varying levels of success probabilities, with some achieving better-than-classical performance across various quantum computing platforms^34–39^. However, these implementations have generally been limited to relatively small values of *S*. For instance, better-than-classical success rates were achieved for $n = 3$^34,36^ and $n = 4$ qubits^38^. The most extensive implementation to date involves $n = 8$ qubits, but this has not yet surpassed classical success probabilities^35^. The study in^39^ demonstrated improved success probabilities for $n = 5$ with a single marked state ($\left| 01011\right\rangle$); however, averaging over multiple marked states ($2^5$) may decrease these probabilities. More recent advancements, such as those reported in^40^, utilized IBM Quantum’s two 7-qubit systems to achieve superior average success probabilities for $n \le 5$ through error suppression techniques. Additional progress in Grover’s algorithm on NISQ devices can be found in^38–45^, reflecting ongoing improvements and innovative approaches. Recently, significant progress towards practical quantum computing using a 127-qubit superconducting quantum processor is demonstrated in^46^, achieving meaningful computational results despite the challenges of noise and lack of fault tolerance. Encouraged by these advancements, our research leverage similar cutting-edge technology, exploring the potential for scalable and efficient GSA implementations in such quantum hardware.

The motivation behind our research lies in the growing interest in practical quantum computing and the necessity to comprehend the experimental feasibility and performance of implementing quantum algorithms on real quantum hardware, especially within the NISQ era. This paper presents a comprehensive study focusing on the experimental implementation and characterization of the GSA on large-scale superconducting quantum computers. Leveraging state-of-the-art quantum hardware, we aim to explore the scalability, performance, and practical challenges associated with deploying Grover’s algorithm in real-world settings.

The remainder of this paper is organized as follows: Section 2 begins with an overview of the experimental setup and implementation details, detailing the procedures and methodologies employed to realize the GSA on the real quantum hardware. In Section 2, we delve into the intricacies of quantum circuit design, oracle construction, and quantum state preparation, laying the groundwork for our experimental investigations. Subsequently, in Section 3, we conduct a thorough characterization of the implemented algorithm, employing ${\mathcal {QST}}$ (quantum state tomography). We design and perform ${\mathcal {QST}}$ experiments, allowing us to assess the algorithm’s performance across different configurations and environmental conditions, including noise-free, noisy, and real quantum hardware. To our knowledge, similar QST experiments have not been conducted on comparable architectures. We analyze key metrics such as algorithm success probability (${\mathcal {ASP}}$), squared statistical overlap (${\mathcal {SSO}}$), and state fidelity ($\mathcal {F}_S$), providing insights into the algorithm’s behavior and its suitability for practical applications. Furthermore, Section 4 includes a detailed analysis and discussions, where we interpret our experimental findings, compare them with theoretical expectations, and explore implications for future research and development in quantum computing. Finally, Section 6 concludes the paper with a summary of our findings and suggestions for future research directions.

## Experimental setup and implementations

### Problem statement

Grover’s algorithm addresses the problem of unstructured search. In this context, an unstructured search problem entails having a set of *S* elements forming a set $\Upsilon = \{\gamma _1, \gamma _2, \dots , \gamma _S\}$, along with a boolean function $f :\Upsilon \rightarrow \{0, 1\}$. The objective is to identify an element $\gamma ^*$ in $\Upsilon$ for which $f(\gamma ^*) = 1$.

For instance, consider a scenario where we are searching for a specific phone number in a directory. The function $f(\gamma )$ evaluates whether a given phone number matches the desired one. The essence of the problem lies in its abstraction, such that any search task can be distilled into an evaluation of a function $f(\gamma )$, where $\gamma$ represents potential search items. Should a particular item $\gamma$ hold the solution, the function returns 1; otherwise, it returns 0. Thus, the fundamental challenge (search problem) is to uncover any such $\gamma ^*$ that yields a result of 1.

Grover’s algorithm embarks on this quest by tackling a classical function ($f(\gamma ): \{0,1\}^n \rightarrow \{0,1\}$), where *n* denotes the bit-size of the search space. In the classical realm, the algorithm’s complexity hinges on the sheer number of times the function $f(\gamma )$ must be interrogated. In the most unfavorable scenario, this involves an exhaustive search through $S-1$ iterations, where $S=2^n$, exhaustively exploring every conceivable option. However, GSA offers a profound departure from this laborious approach, promising a remarkable quadratic acceleration. Specifically, this signifies that the algorithm can ascertain the sought-after solution with a mere $\mathcal {O}(\sqrt{S})$ evaluations, a stark contrast to the linear *S* evaluations demanded by classical methods^17^.

### Methodology

Grover’s algorithm not only revolutionizes the speed at which search tasks are accomplished but also epitomizes the transformative potential of quantum computing in navigating complex computational landscapes with unprecedented efficiency. Its methodology comprises several pivotal stages: initialization, marking, amplification, and measurement, as depicted in Figure 1. The algorithm initiates with an input state $\left| \zeta _0\right\rangle = \sum _{\chi =0}^{S-1} \left| \chi \right\rangle \rightarrow \left| 0\right\rangle ^{\otimes n}$, where it establishes a superposition of all potential search states, $H^{\otimes n} \left| \zeta _0\right\rangle \rightarrow \left| \zeta _1\right\rangle = \frac{1}{\sqrt{S}}\sum _{\chi =0}^{S-1} \left| \chi \right\rangle$, laying the groundwork for quantum parallelism. Subsequently, during the marking phase, an oracle function ($\mho _f:\left| \chi \right\rangle \rightarrow (-1)^{f(\chi )} \left| \chi \right\rangle$) is employed to identify and mark the target state $\left| \chi ^*\right\rangle$ or states within the superposition. These oracle functions alter the sign of the amplitude of the marked state(s). Mathematically, for a single-search result scenario, it can be represented as $\left| \zeta _2\right\rangle = -\frac{1}{\sqrt{S}} \left| \chi ^*\right\rangle + \frac{1}{\sqrt{S}} \sum _{\chi =0,\chi \ne \chi ^*}^{S-1} \left| \chi \right\rangle$. This marking process is crucial as it enables the algorithm to concentrate computational resources on the desired outcome.

The heart of Grover’s algorithm lies in the amplification phase (see Figure 1), where a Grover operator ($\mathcal{G}\mathcal{O}$) is iteratively applied to boost the probability amplitude of the marked state(s) while simultaneously diminishing the amplitudes of non-marked states. The $\mathcal{G}\mathcal{O}$ involves reflections about the mean ($\mu _G = \frac{1}{S} \sum _{\chi =0}^{S-1} \varpi _{\chi }$) so that $\left| \zeta _3\right\rangle = -\varpi _{\chi ^*} \left| \chi ^*\right\rangle + \varpi _{\chi } \sum _{\chi =0,\chi \ne \chi ^*}^{S-1} \left| \chi \right\rangle$, and inversions about the marked state, $\left| \zeta _4\right\rangle = (2 \mu _G + \varpi _{\chi ^*}) \left| \chi ^*\right\rangle + (2 \mu _G - \varpi _{\chi }) \sum _{\chi =0,\chi \ne {\chi ^*} }^{S-1} \left| \chi \right\rangle$, where $\varpi _{\chi }$ denotes the amplitude for the basis state $\left| \chi \right\rangle$. This iterative process is pivotal in achieving the algorithm’s remarkable time complexity of $\mathcal {O}(\sqrt{S})$, representing a quadratic speedup over classical search algorithms. Finally, the algorithm concludes with a measurement stage, wherein the quantum state is measured, yielding the marked item(s) with high probability. The correctness of the algorithm is validated by assessing the probability of measuring the marked state.

### Design and execution of quantum oracles

**Table 1:**
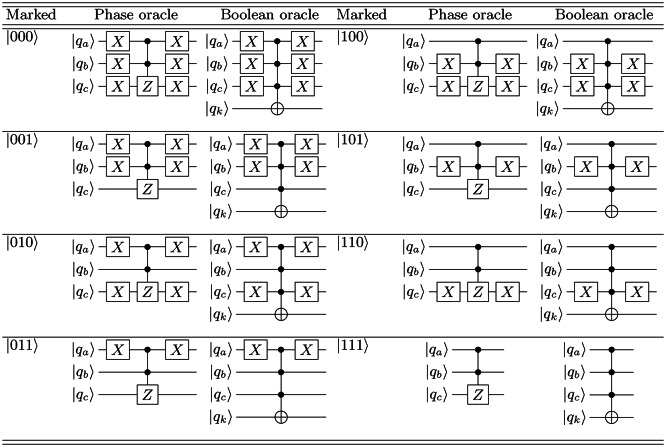
Single-solution GSA phase oracles and their corresponding boolean oracles, for all eight single-marked states employed in the 3-qubit GSA^34^. Illustrated using conventional notation for quantum circuit diagrams, where $q_k$ denotes an ancillary qubit.

**Table 2:**
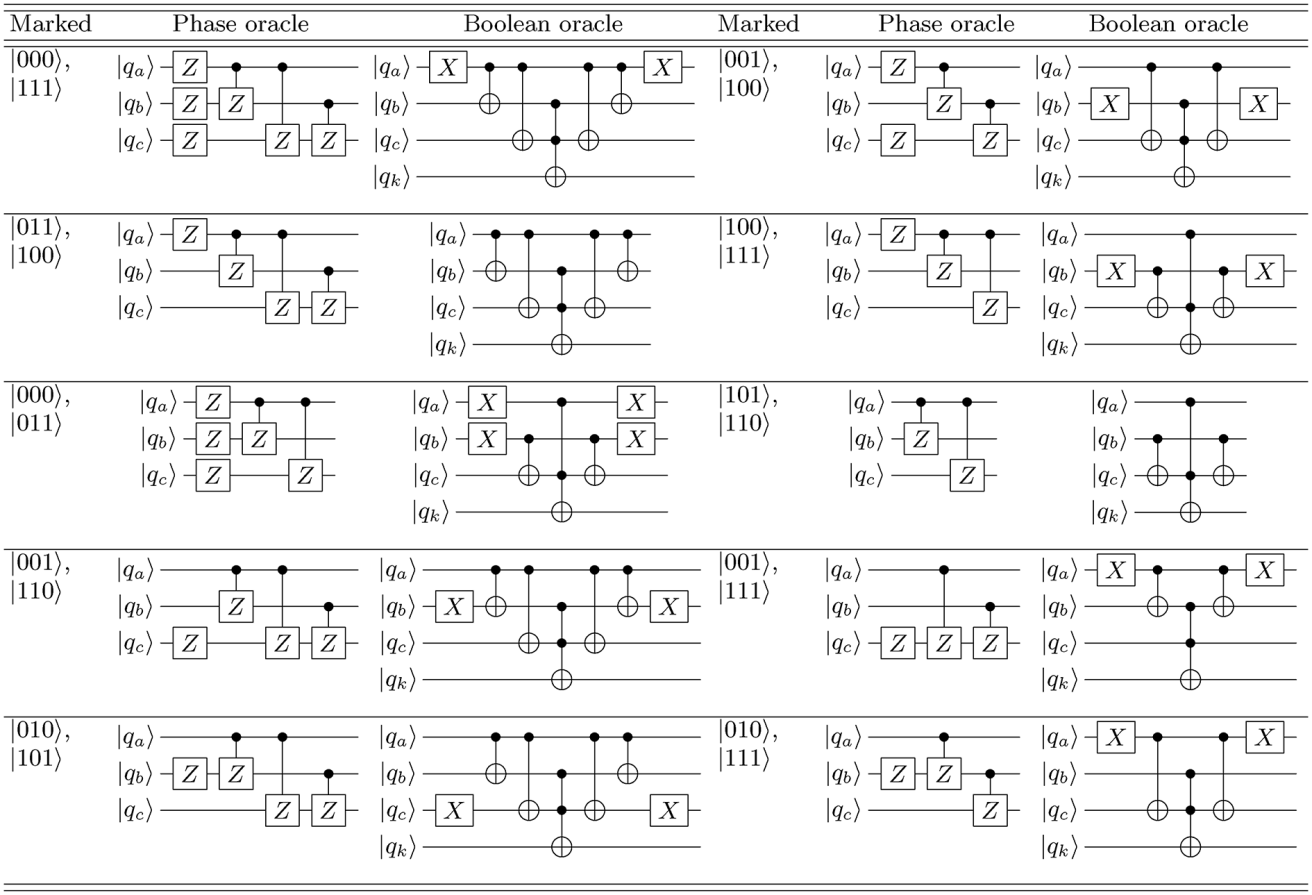
A random sample of two-solution GSA phase oracles and their corresponding boolean oracles. In our experimental investigation, we analyze the performance of the GSA with nine distinct 2-search phase oracles, each uniquely designed to mark two states within the search space. Illustrated using conventional notation for quantum circuit diagrams.

Here, we implement the GSA using state-of-the-art scalable superconducting quantum computers, utilizing $n = 3$ qubits, which corresponds to a search database of size $S = 2^n = 8$. Table 1 illustrates the phase oracles alongside their corresponding boolean oracles for all eight single-marked states employed in the 3-qubit GSA. These marked states are: $\left| 000\right\rangle , \, \left| 001\right\rangle$, $\left| 010\right\rangle , \, \left| 011\right\rangle$, $\left| 100\right\rangle , \, \left| 101\right\rangle$, $\left| 110\right\rangle , \, \text {and} \left| 111\right\rangle$. Additionally, we scrutinize the performance of the GSA with nine distinct two-search phase oracles (as depicted in Table 2), each uniquely designed to mark two states within the search space. The marked states include: $\left| 000\right\rangle -\, \left| 111\right\rangle$, $\left| 001\right\rangle -\, \left| 100\right\rangle$, $\left| 011\right\rangle -\, \left| 100\right\rangle$, $\left| 010\right\rangle -\, \left| 111\right\rangle$, $\left| 000\right\rangle -\, \left| 110\right\rangle$, $\left| 010\right\rangle -\, \left| 101\right\rangle$, $\left| 101\right\rangle -\, \left| 110\right\rangle$, $\left| 101\right\rangle -\, \left| 111\right\rangle$, and $\left| 100\right\rangle -\, \left| 111\right\rangle$. In our experimental setups, we employ the GSA with phase oracles, a technique previously verified in other experimental configurations. It is noteworthy that both phase and boolean oracles exhibit mathematical equivalence^47^.

The likelihood of detecting the designated state following *k* iterations of the $\mathcal{G}\mathcal{O}$ is a crucial metric for evaluating the algorithm’s efficiency. This likelihood is articulated by the expression:

1 $$\begin{aligned} \mathcal {P}(\left| \chi ^*\right\rangle ) = k \cdot \left( \left[ \frac{\pounds -2k}{\pounds } + \frac{2(\pounds -k)}{\pounds } \right] \frac{1}{\sqrt{\pounds }} \right) ^2 \end{aligned}$$

This formulation arises from the amplitude amplification process^18^, delineating the probability amplitude alterations occurring at each iteration.

In the case of a single-solution algorithm with $k = 1$ iteration in GSA, the algorithmic probability of measuring the correct state after one iteration results in approximately $78.125\%$. This probability is significantly higher than the probability achieved by the optimal classical search strategy, which consists of a single query followed by a random guess in case of failure. In the classical strategy, the probability is calculated as:

2 $$\begin{aligned} \mathcal {P}_{\text {classical}} = \frac{k}{\pounds } + \left( 1 - \frac{k}{\pounds }\right) \cdot \frac{k}{\pounds -1} \end{aligned}$$

For this case, it equals $25\%$, where *k* represents the number of correct answers (1 in this case) and $\pounds$ represents the total number of possible answers^34^. This comparison highlights the quantum advantage of GSA over classical search strategies, demonstrating its superior efficiency in finding solutions with fewer queries, especially in scenarios with a single correct answer.

### Quantum circuits construction

The complexity of circuit transpilation increases with problem size, particularly due to the need for decomposing multi-qubit gates. Implementing the *n*-qubit GSA necessitates the use of the *n*-qubit controlled-phase gate, denoted $\Gamma _{n-1}(Z)$. This multi-qubit gate is critical for both the oracle and the amplitude amplification steps of the algorithm^40^. The $\Gamma _{n-1}(Z)$ gate can be realized through the decomposition of the *n*-qubit Toffoli gate, denoted $\Gamma _{n-1}(X)$. This decomposition is resource-intensive. Consequently, circuits with multiple Toffoli gates tend to have significant depth, which can increase their susceptibility to noise and gate errors.

In practical quantum devices, such as NISQ superconducting devices, the challenge of implementing multi-controlled NOT gates is compounded by limited qubit connectivity, making such decomposition’s into two-qubit gates more complex. For instance, each three-qubit Toffoli gate ($\Gamma _{2}(X)$ or CCX) in a quantum circuit can require up to *six* CNOT gates in addition to several single-qubit gates^47^. However, this decomposition assumes a fully connected quantum architecture, which is not always available in practical quantum devices. Alternative circuit decomposition, for linear connected three qubits, such as the decomposition of the three-qubit $\Gamma _{2}(Z)$ gate into *eight* CNOT gates have been explored in^38–40^. This scheme, utilizing specific relative phase Toffoli gates^48^ can be generalized to implement the n-qubit GSA^40^.

In our experiments, the quantum hardware leveraged a two-qubit gate, known as the ECR (Echoed Cross-Resonance) gate, rather than using the conventional CNOT gate. In our implementations, the CCX gate is decomposed into *nine* ECR gates^49^, see Eq. (3). Leveraging the ECR gate’s advanced capabilities, including enhanced entanglement and error mitigation, which are particularly well-suited for the specific needs of our experiments.3

The ECR gate is a maximally entangling gate, functions similarly to the CNOT gate through a distinct mechanism that is optimized for the specific architecture of the hardware^50^. In particular, the $ECR^{q_0}_{q_1}$ gate implements the operation $(IX-XY)/\sqrt{2}$, where *I* is the identity matrix, *X* and *Y* are Pauli matrices, with $R_z$ are single-qubit rotations about the *z* axis. This echoing process improves the gate’s performance in practical experiments by mitigating errors and preserving the fidelity of the quantum state. See Eq. (4) for ECR’s matrix representations.

4 $$\begin{aligned} ECR ^{q_0}_{q_1} = \frac{1}{\sqrt{2}} \begin{pmatrix} 0 & 1 & 0 & i \\ 1 & 0 & -i & 0 \\ 0 & i & 0 & 1 \\ -i & 0 & 1 & 0 \end{pmatrix}, \quad ECR ^{q_1}_{q_0} = \frac{1}{\sqrt{2}} \begin{pmatrix} 0 & 0 & 1 & i \\ 0 & 0 & i & 1 \\ 1 & -i & 0 & 0 \\ -i & 1 & 0 & 0 \end{pmatrix} \end{aligned}$$

### Results overview

The ${\mathcal {ASP}}$ encapsulates the probability of successfully identifying the marked state as the conclusive result of an experiment. In the case of a two-solution algorithm, ${\mathcal {ASP}}$ is determined by aggregating the probabilities associated with observing each of the two marked states. Meanwhile, the ${\mathcal {SSO}}$ offers a quantitative measure of the extent to which observed and expected populations overlap across all states. The ${\mathcal {SSO}}$ is calculated as follows:

5 $$\begin{aligned} {\mathcal {SSO}} = \left( \sum _{m=0}^{M} \sqrt{\xi _m \cdot \mathcal {M}_m} \right) ^2 \end{aligned}$$

Here, $\xi _m$ represents the expected population for each state *m*, while $\mathcal {M}_m$ signifies the measured population for each state *m*, and *M* denotes the total number of states. This formulation of ${\mathcal {SSO}}$ is often used in various fields, including population studies, where it quantifies the similarity or overlap between observed and expected population distributions across different categories or states^51^.

In our investigation, we delved into executions of the GSA for both single-solution and two-solution scenarios on a 3-qubit database, exploring diverse environmental conditions such as noisy environments and leveraging real quantum computers provided by IBM Quantum^52^. Specifically, we employed three of IBM’s 127-qubit superconducting quantum computers: *ibm_sherbrook*. *ibm_osaka*, and *ibm_kyoto*. These quantum machines are equipped with Eagle r3 quantum processors, boasting capabilities of executing a maximum of 300 circuits and 100,000 shots. For further insights, the key specifications and qubit characteristics of these 127-qubit quantum computers, including error rates for individual gates and readout, as well as their basis gates, are meticulously outlined in Section 5.

While acknowledging the theoretical optimality of GSA, which suggests a runtime of $\mathcal {O}(\sqrt{S})$ iterations to locate the marked state within a search space of size *S*, our experimental implementations were tailored to address the practical realities of quantum hardware, while still showcasing the algorithm’s effectiveness. Thus, we iterate the $\mathcal{G}\mathcal{O}$ ten times for each oracle, guided by the prevalent challenges posed by errors and noise in real-world quantum hardware, particularly in the NISQ era.

**Figure 2:**
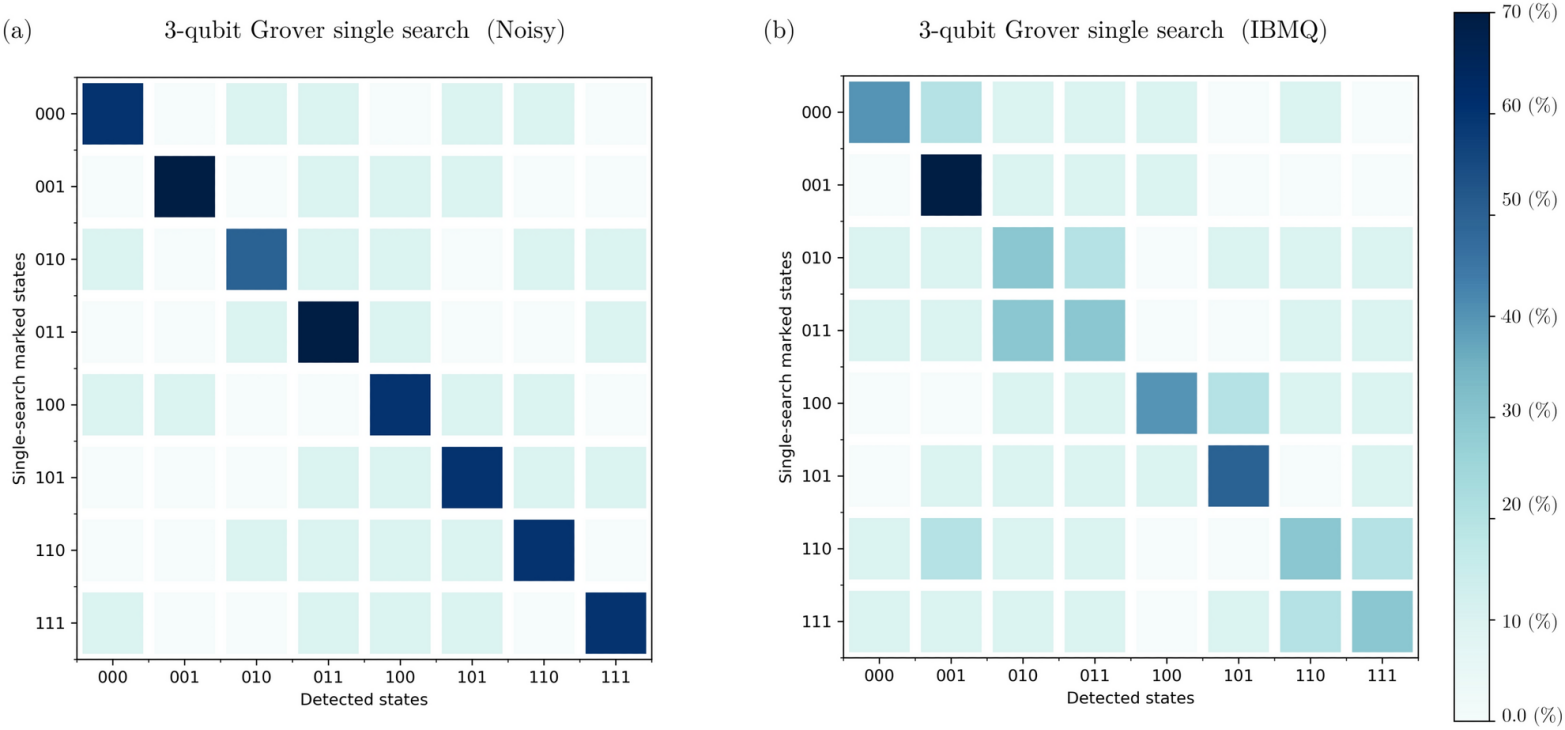
Results obtained from executing the GSA for single-solution scenarios on a 3-qubit database (000, 001, 010, 011, 100, 101, 110, 111) in various environments. The left side presents data from algorithm execution in a noisy environment, while the right side displays data from execution on IBM Quantum’s real quantum computers. The graphs illustrate the probability distribution for each output state. We observed a median ${\mathcal {ASP}}$ of 76.79% in the noisy execution and 44.80% on the IBM quantum computers. Additionally, we obtained a median ${\mathcal {SSO}}$ of 82.49% in the noisy environment and 72.63% on real IBM quantum computers. All percentages are calculated relative to the expected state $\left| \psi _E\right\rangle _\text {Single}$, defined as $\left| \psi _E\right\rangle _\text {Single} = \frac{5}{4\sqrt{2}} \left| \chi ^*\right\rangle +\frac{1}{4\sqrt{2}} \sum _{\chi \ne \chi ^*} \left| \chi \right\rangle$, where $\left| \chi ^*\right\rangle$ represents the single marked state. The $\left| \,\right\rangle$ notation was omitted from the figures for simplicity.

For the single-solution scenarios, we observed intriguing outcomes (see Figure 2). In the presence of noise, our analysis revealed an average ${\mathcal {ASP}}$ of $78.39\%$, indicating the algorithm’s capability to consistently identify the correct solution amidst environmental disturbances. However, when executed on IBM Quantum’s real quantum computers, the ${\mathcal {ASP}}$ decreased to $51.19\%$, underscoring the challenges encountered in practical quantum computing environments. Furthermore, our investigation into the ${\mathcal {SSO}}$ metrics provided deeper insights. In noisy environments, we recorded an average ${\mathcal {SSO}}$ of $82.358\%$, reflecting the algorithm’s ability to closely approximate the target state despite environmental noise. Conversely, on real IBM quantum computers, the average ${\mathcal {SSO}}$ decreased to $73.12\%$, indicating the degree of deviation from the expected state.

**Figure 3:**
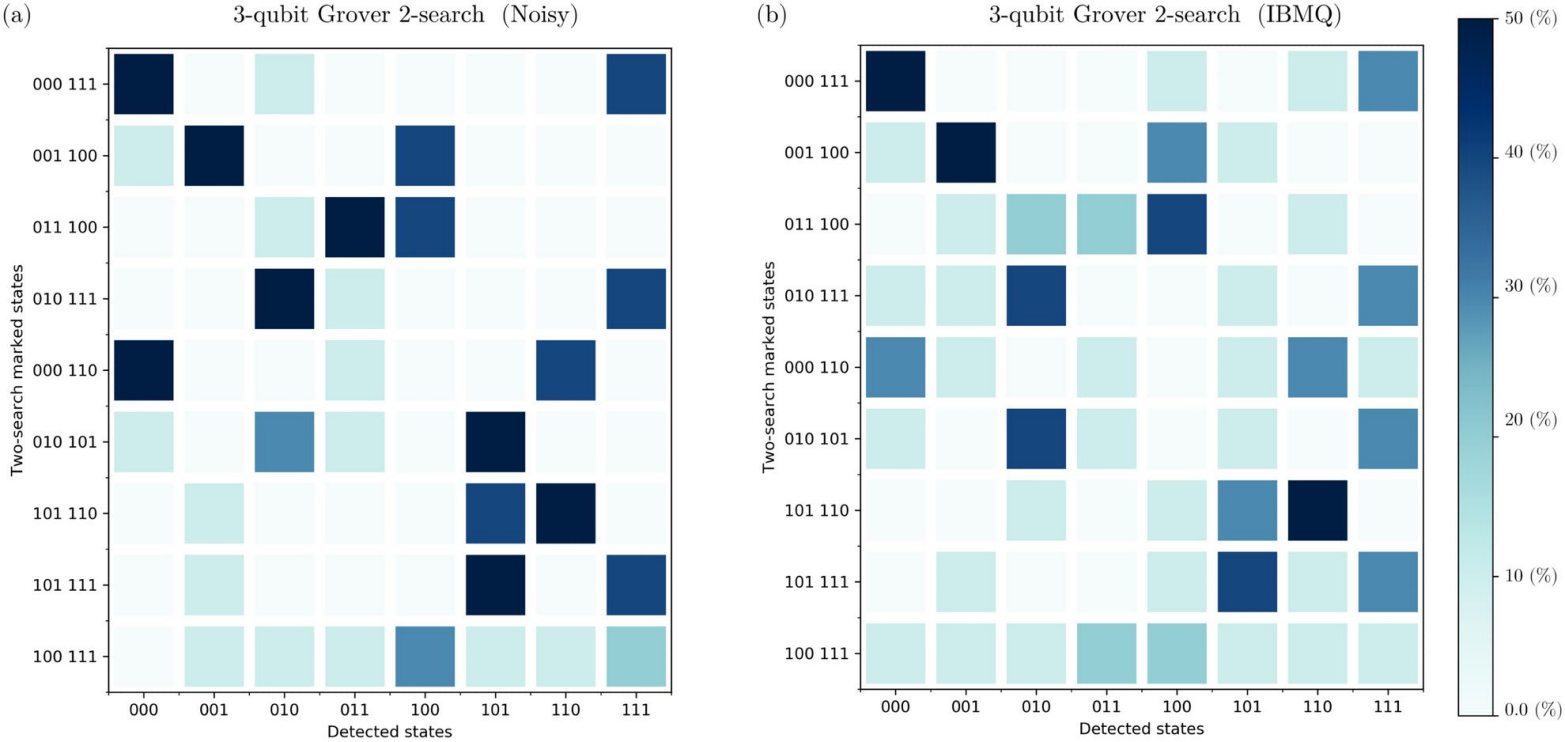
Results derived from executing the GSA for nine two-solution scenarios in various environments on a 3-qubit database. The data from algorithm execution in a noisy environment is presented on the left, while data from execution on IBM Quantum’s real quantum computers is displayed on the right. The graphs depict the probability distribution for each output state. We observed an average ${\mathcal {ASP}}$ of 84.44% in the noisy execution and 64.44% on the IBM quantum computers. Additionally, we obtained an average ${\mathcal {SSO}}$ of 84.03% in the noisy environment and 63.10%, on real IBM quantum computers. All percentages are calculated relative to the expected state $\left| \psi _E\right\rangle _\text {Multi}$, defined as $\left| \psi _E\right\rangle _\text {Multi} = \frac{1}{\sqrt{2}} \left| \chi ^*_1\right\rangle +\frac{1}{\sqrt{2}} \left| \chi ^*_2\right\rangle$, where $\left| \chi ^*_1\right\rangle$ and $\left| \chi ^*_2\right\rangle$ represents the two marked states.

Transitioning to the two-solution scenarios, our findings revealed notable trends (see Figure 3). In noisy executions, the algorithm displayed a robust performance, achieving an average ${\mathcal {ASP}}$ of $84.44\%$. However, on IBM Quantum’s real quantum computers, the ${\mathcal {ASP}}$ reduced to $64.44\%$, suggesting the impact of practical constraints on algorithmic efficacy. Examining the ${\mathcal {SSO}}$ values in these scenarios provided additional insights. In noisy environments, the average ${\mathcal {SSO}}$ was $84.03\%$, indicating a satisfactory overlap with the expected state despite noise. Conversely, on real IBM quantum computers, the average ${\mathcal {SSO}}$ decreased to $63.10\%$, highlighting the challenges encountered in achieving precise outcomes in practical quantum computing in the NISQ era.

## Experimental characterization of the Grover search algorithm

### ${\mathcal {QST}}$ experiments

${\mathcal {QST}}$ plays a pivotal role in the development and validation of quantum technologies by providing a comprehensive characterization of quantum states^53–55^. It allows extracting essential information about the state of a quantum system, enabling precise assessment of quantum operations’ fidelity and performance^53,54,56–60^. By reconstructing quantum states experimentally, ${\mathcal {QST}}$ helps identify and quantify sources of errors, assess the effectiveness of error mitigation techniques, and verify the fidelity of quantum gates and algorithms^61–64^. Moreover, it serves as a crucial tool for benchmarking and calibrating quantum devices, facilitating progress towards achieving reliable and scalable quantum computation and communication protocols^65,66^.

Here, we undertake a comprehensive exploration through five ${\mathcal {QST}}$ experiments to meticulously evaluate the behavior and efficiency of our implemented algorithm under varied conditions. Specifically, we conduct two ${\mathcal {QST}}$ experiments for the GSA employing single search oracles-$\left| 010\right\rangle$ and $\left| 101\right\rangle$. Moreover, three additional ${\mathcal {QST}}$ experiments are meticulously performed for the GSA utilizing two search oracles–$\left| 000\right\rangle -\left| 111\right\rangle$, $\left| 101\right\rangle -\left| 110\right\rangle$, and $\left| 101\right\rangle -\left| 111\right\rangle$. These experiments are meticulously executed across three distinct environments: a pristine noise-free setting, a simulated noisy environment, and on IBM Quantum’s tangible quantum computers. For each setting, we measured the state fidelity of the output states produced by the algorithm. The state fidelity represents the similarity between the output states and the desired target states, $0 \le {\mathcal {F}}_{\mathcal {S}} \le 1$, with higher fidelity values indicating better performance^47,67^.

**Figure 4:**
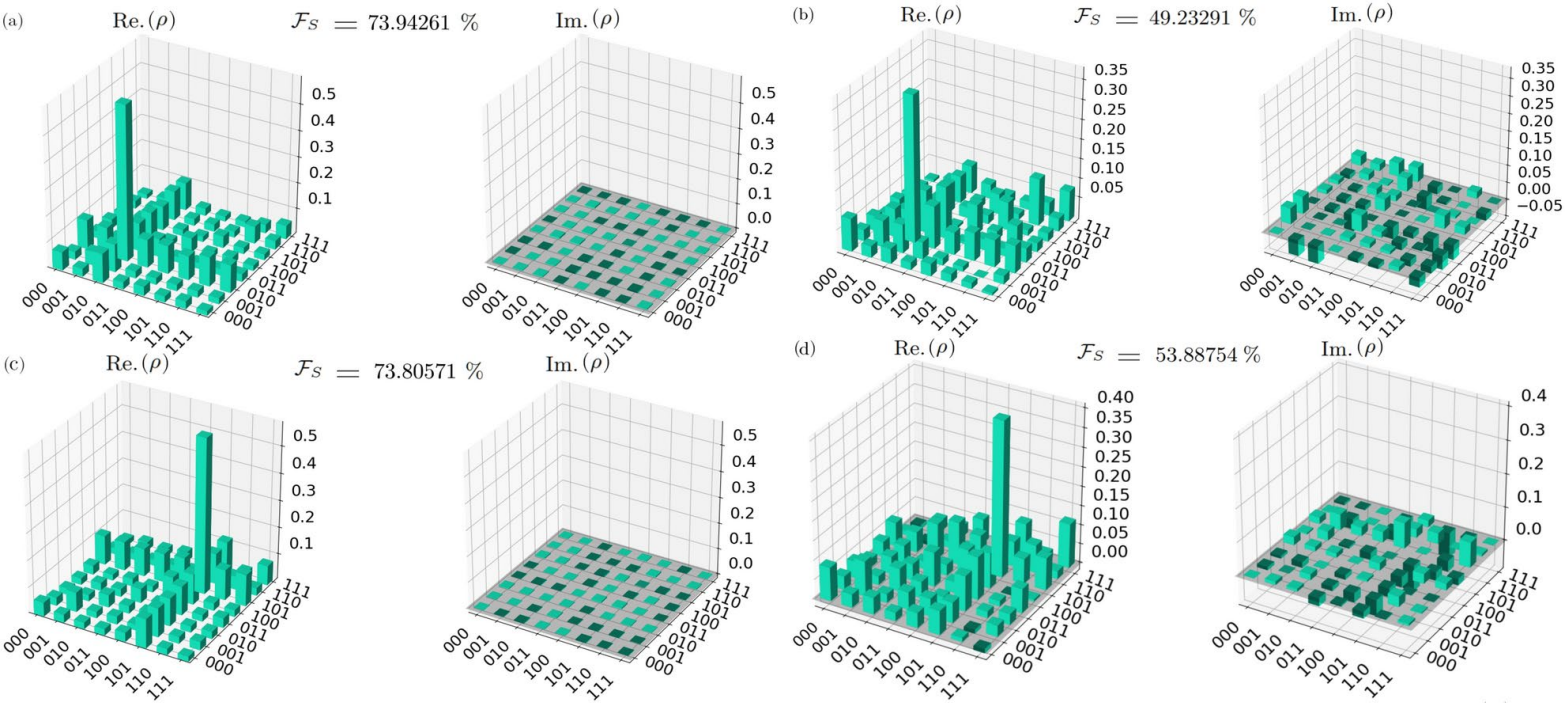
Results from ${\mathcal {QST}}$ experiments on the GSA with single-search oracles in different environments. (**a**) Real (Re.$(\rho )$) and imaginary (Im.$(\rho )$) parts of the density matrix obtained from ${\mathcal {QST}}$ experiments for the single-search marked state $\left| 010\right\rangle$, executed with 7797 repeated shots in a noisy environment, (**b**) and 1024 repeated shots on IBM Quantum’s 127-qubit superconducting quantum computer *ibm_osaka*, yielding state fidelities ($\mathcal {F}_S$) of 73.9426% and 49.2329%, respectively. (**c**) Re.$(\rho )$ and Im.$(\rho )$ parts of the density matrix obtained from ${\mathcal {QST}}$ experiments for the single-search marked state $\left| 101\right\rangle$, executed with 7797 repeated shots in a noisy environment, (**d**) and 7797 repeated shots on *ibm_osaka*, yielding $\mathcal {F}_S$ of 73.8057% and 53.8875%, respectively.

**Figure 5:**
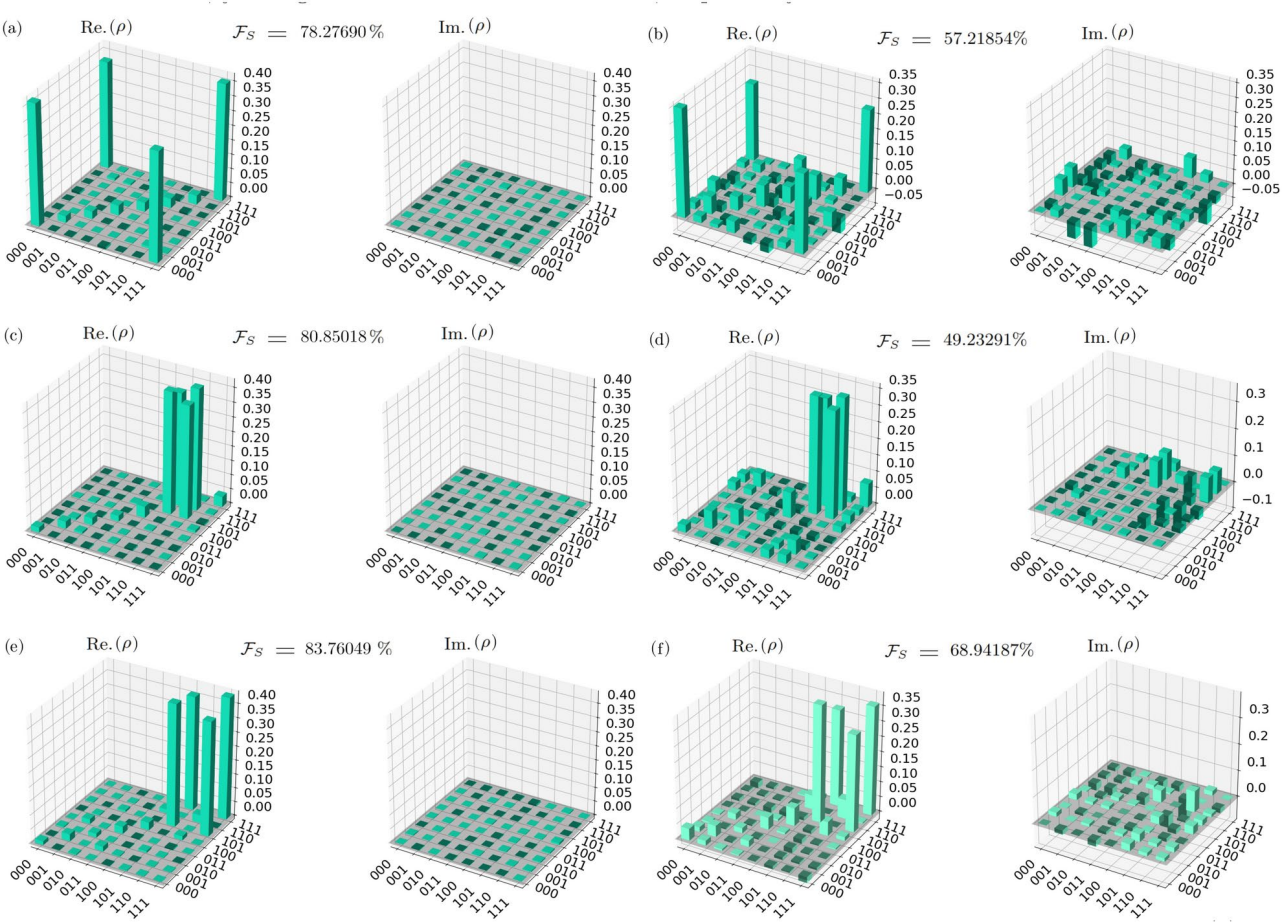
Results from ${\mathcal {QST}}$ experiments of the GSA employing two-search oracles in different environments. (**a**) Real (Re.$(\rho )$) and imaginary (Im.$(\rho )$) parts of the density matrix obtained from ${\mathcal {QST}}$ experiments for the two-search marked states $\left| 000\right\rangle$ and $\left| 111\right\rangle$, executed with 7797 repeated shots in a noisy environment, (**b**) and 7797 repeated shots on IBM Quantum’s 127-qubit superconducting quantum computer *ibm_osaka*, yielding state fidelities ($\mathcal {F}_S$) of 78.2769% and 57.2185%, respectively. (**c**) Re.$(\rho )$ and Im.$(\rho )$ parts of the density matrix obtained from ${\mathcal {QST}}$ experiments for the two-search marked states $\left| 101\right\rangle$ and $\left| 110\right\rangle$, executed with 7797 repeated shots in a noisy environment, (**d**) and 1024 repeated shots on *ibm_osaka*, yielding $\mathcal {F}_S$ of 80.8501% and 49.2329%, respectively. (**d**) Re.$(\rho )$ and Im.$(\rho )$ parts of the density matrix obtained from ${\mathcal {QST}}$ experiments for the two-search marked states $\left| 111\right\rangle$ and $\left| 101\right\rangle$, executed with 7797 repeated shots in a noisy environment, (**e**) and 1024 repeated shots on *ibm_osaka*, yielding $\mathcal {F}_S$ of 83.7605% and 68.9419%, respectively.

In the pursuit of authenticity, when executing our experiments on genuine quantum computers, we meticulously perform ${\mathcal {QST}}$ experiments for the GSA with single-marked states $\left| 010\right\rangle$ and $\left| 101\right\rangle$, employing 1024 and 7779 shots, respectively. The resultant state fidelities ${\mathcal {F}}_{\mathcal {S}}$ are found to be 49.23291% and 53.88754%, respectively.  Additionally, for ${\mathcal {QST}}$ experiments involving two-marked states (two search oracles)–$\left| 000\right\rangle -\left| 111\right\rangle$, $\left| 101\right\rangle -\left| 110\right\rangle$, and $\left| 101\right\rangle -\left| 111\right\rangle$–we replicate the experiments using 7779, 1024, and 1024 shots, respectively. The corresponding state fidelities are observed to be 57.21854%, 49.23291%, and 68.94187%, respectively. The choice of varying numbers of shots for the ${\mathcal {QST}}$ experiments was employed to ensure sufficient statistical sampling and improve the reliability of the results. The outcomes gleaned from ${\mathcal {QST}}$ experiments of the GSA on real quantum computers, utilizing both single-search oracles and two-search oracles, are meticulously presented in Figures 4 and 5, respectively.

Upon examining the results across the three distinct environments, a notable degradation in performance is observed as we transition from the noise-free setting (mean ${\mathcal {F}}_{\mathcal {S}} = 99.38 \%$) to the noisy setting (mean ${\mathcal {F}}_{\mathcal {S}} = 78.13 \%$), and further to the real quantum computer setting (mean ${\mathcal {F}}_{\mathcal {S}} = 54.32 \%$). In our ${\mathcal {QST}}$ experiments conducted on a real superconducting quantum computer for the GSA with different oracles, with a mean ${\mathcal {F}}_{\mathcal {S}}$ of $54.32 \%$ and a standard deviation of 0.099, the algorithm demonstrates a moderate level of consistency in generating quantum states resembling the target state. These findings suggest that while the GSA shows promise in surpassing classical random search strategies, there remains room for improvement to achieve higher and more consistent fidelity. Further experimental investigation could elucidate potential avenues for refinement, ultimately advancing the algorithm’s efficacy in quantum search applications.

## Analysis and discussions

In this section, following the collection of experimental data, we performed a comprehensive statistical analysis. Our aim was to derive meaningful conclusions regarding the performance of the GSA across various settings, focusing on aspects such as ${\mathcal {ASP}}$, ${\mathcal {SSO}}$, state fidelity $\mathcal {F}_S$, and to assess the influence of noise and other factors on its effectiveness.

### ${\mathcal {ASP}}$ analysis

The statistical tests conducted offer critical insights into the ${\mathcal {ASP}}$ of a 3-qubit GSA executed on IBM Quantum’s real quantum computers, particularly in estimating the population of all 2-marked states oracles within the 3-qubit space (see Tables 3 and 4). The mean ${\mathcal {ASP}}$ of 64.44% provides a central measure of the algorithm’s performance in identifying desired states within the quantum system, while the standard deviation of 16.67 offers an indication of the variability in ${\mathcal {ASP}}$ values around the mean. The subsequent one-sample t-test comparing the observed mean ${\mathcal {ASP}}$ against a hypothesized population mean yields a non-significant* p*-value of 0.99, indicating that the observed mean ${\mathcal {ASP}}$ is likely representative of the population mean. This suggests that the algorithm’s performance, as observed on IBM Quantum’s real quantum computers, aligns closely with theoretical expectations or benchmark values. Similarly, the hypothesis test for variance, with a* p*-value of 0.868, suggests no significant difference in the variability of ${\mathcal {ASP}}$ between the observed sample and the hypothetical population. Thus, the variability in ${\mathcal {ASP}}$ observed in the sample is deemed consistent with that of the population. Overall, these results underscore the reliability and validity of the observed ${\mathcal {ASP}}$ values in estimating the population of 2-marked states within the 3-qubit space.

### ${\mathcal {SSO}}$ analysis

The statistical tests conducted on the ${\mathcal {SSO}}$ for a 3-qubit GSA executed on IBM Quantum’s real quantum computers provide reliable insights into estimating population parameters for all 2-marked states oracles within the 3-qubit space (see Table 3 and 4). The mean ${\mathcal {SSO}}$ value of 63.10 represents the average squared statistical overlap between observed two-search result oracles, with a standard deviation of 17.07 indicating variability around this mean. A one-sample t-test with a 95% confidence interval (CI), yielding a non-significant* p*-value of 0.99, suggests that the observed mean ${\mathcal {SSO}}$ is representative of the population mean. Additionally, the 95% confidence intervals for variance offer insights into the precision of estimated variability within the population. The hypothesis test for variance, with a* p*-value of 0.867, indicates no significant difference in variability between the observed sample and the hypothetical population. These results underscore the accuracy and reliability of the observed ${\mathcal {SSO}}$ values in estimating population parameters.

**Table 3:** Comparative analysis of ${\mathcal {SSO}}$ and ${\mathcal {ASP}}$ for all 8 ($2^n$) single-search result oracles in the GSA. The experiments are conducted under diverse conditions: noise-free, noisy, and on IBM Quantum’s 127-qubit superconducting quantum computers$^{1}$.

Marked State	${\mathcal {SSO}}$ (Noisy setting)	${\mathcal {SSO}}$ (IBM Quantum)	${\mathcal {ASP}}$ (Noisy setting)	${\mathcal {ASP}}$ (IBM Quantum)
(1) 000	82.49 (%)	74.25 (%)	76.79 (%)	51.20 (%)
(2) 001	82.30 (%)	82.30 (%)	89.59 (%)	89.59 (%)
(3) 010	81.81 (%)	71.01 (%)	64.00 (%)	38.39 (%)
(4) 011	82.30 (%)	64.73 (%)	89.59 (%)	38.39 (%)
(5) 100	82.49 (%)	74.25 (%)	76.79 (%)	51.20 (%)
(6) 101	82.49 (%)	81.81 (%)	76.79 (%)	64.00 (%)
(7) 110	82.49 (%)	65.60 (%)	76.79 (%)	38.39 (%)
(8) 111	82.49 (%)	71.01 (%)	76.79 (%)	38.39 (%)
Median	82.49 (%)	72.63 (%)	76.79 (%)	44.80 (%)
StDev	0.237	6.53	8.20	18.10
Mean	82.358 (%)	73.12 (%)	78.39 (%)	51.19 (%)
SE Mean$^{2}$	0.0839	2.31	2.90	6.40

$^{1}$Data from the noise-less environment is not presented here, as it closely approaches 99.99%. For a comparison of the GSA performance across various environments, see Section 5. $^2$The standard error of the mean ($\text {SE Mean}=\text {StDev}/\sqrt{S}$)

**Table 4:** Comparative Analysis of ${\mathcal {SSO}}$ and ${\mathcal {ASP}}$ for nine two-search result oracles in the GSA. The experiments are conducted under various conditions: noise-free, noisy, and on IBM Quantum’s 127-qubit superconducting quantum computers. Additionally, the analysis incorporates hypothesis testing and $95\%$ CI for both the population mean ($\mu$) and variance ($\sigma ^2$).

Two-Marked State	${\mathcal {SSO}}$ (Noisy setting)	${\mathcal {SSO}}$ (IBM Quantum)	${\mathcal {ASP}}$ (Noisy setting)	${\mathcal {ASP}}$ (IBM Quantum)
(1) 000, 111	89.72 (%)	78.72 (%)	90.00 (%)	80.00 (%)
(2) 001, 100	89.72 (%)	78.72 (%)	90.00 (%)	80.00 (%)
(3) 011, 100	89.72 (%)	58.28 (%)	90.00 (%)	60.00 (%)
(4) 010, 101	89.72 (%)	69.64 (%)	90.00 (%)	70.00 (%)
(5) 000, 110	89.72 (%)	60.00 (%)	90.00 (%)	60.00 (%)
(6) 010, 101	78.73 (%)	45.00 (%)	80.00 (%)	50.00 (%)
(7) 101, 110	89.72 (%)	78.72 (%)	90.00 (%)	80.00 (%)
(8) 101, 111	89.72 (%)	69.64 (%)	90.00 (%)	70.00 (%)
(9) 100, 111	49.49 (%)	29.14 (%)	50.00 (%)	30.00 (%)
Mean	84.03 (%)	63.10 (%)	84.44 (%)	64.44 (%)
StDev	13.45	17.07	13.33	16.67
SE Mean	4.48	5.69	4.44	5.56
t-test (95% CI)	(73.69, 94.37)	(49.97, 76.22)	(74.20, 94.69)	(51.63, 77.26)
Hypothesis tests (Mean$^1$)	*P*-value: 1.00	0.99	0.99	0.99
95% CI StDev	(9.10, 25.8)	(11.5, 32.7)	(9.00, 25.5)	(11.3, 31.9)
95% CI Variance	(83, 664)	(133, 1070)	(81, 625)	(127, 1019)
Hypothesis tests (Variance$^2$)	*P*-value: 0.866	0.867	0.866	0.868

$^1$The null and alternative hypothesis for the mean are: $\mu =84.03$ vs $\mu \ne 84.03$, $\mu =63.10$ vs $\mu \ne 63.10$, $\mu =84.44$ vs $\mu \ne 84.44$, and $\mu =64.44$ vs $\mu \ne 64.44$, respectively. $^2$The null and alternative hypothesis for the variance are: $\sigma =13.45$ vs $\sigma \ne 13.45$, $\sigma =17.07$ vs $\sigma \ne 17.07$, $\sigma =13.33$ vs $\sigma \ne 13.33$, and $\sigma =16.67$ vs $\sigma \ne 16.67$, respectively

**Table 5:** Analysis of state fidelity ($\mathcal {F}_S$) derived from ${\mathcal {QST}}$ experiments conducted on the GSA, encompassing both single-search and two-search oracles. The experiments are performed across various environments: Noise-free, Noisy, and on IBM Quantum’s 127-qubit superconducting quantum computers. Furthermore, the analysis includes hypothesis testing and $95\%$ CI for the population mean ($\mu$) and variance ($\sigma ^2$).

Marked State	$\mathcal {F}_S$ ${\mathcal {QST}}$ (Noise-free)	$\mathcal {F}_S$ ${\mathcal {QST}}$ (Noisy settings)	$\mathcal {F}_S$ ${\mathcal {QST}}$ (IBM Quantum)
Single-search (010)	0.9946673	0.7394261	0.4923291
Single-search (101)	0.9946291	0.7380571	0.5388754
Two-search (000, 111)	0.9956002	0.7827690	0.5721854
Two-search (101, 110)	0.9922922	0.8085018	0.4923291
Two-search (111, 101)	0.9921588	0.8376049	0.6894187
Mean	0.993870	0.7813	0.5432
StDev	0.001551	0.0434	0.0990
SE Mean	0.000694	0.01194	0.0443
t-test (95% CI)	(0.9919, 0.9957)	(0.7274, 0.8352)	(0.4205, 0.6661)
Hypothesis tests (Mean$^1$)	*P*-value: 0.999	0.999	1.000
95% CI StDev	(0.00093, 0.00446)	(0.0260, 0.1247)	(0.05930, 0.02845)
95% CI Variance	(0.000001, 0.00002)	(0.00068, 0.01556)	(0.00352, 0.08093)
Hypothesis tests (Variance$^2$)	*P*-value: 0.812	0.812	0.812

$^1$The null and alternative hypothesis for the mean are: $\mu =0.9938$ vs $\mu \ne 0.9938$, $\mu =0.7813$ vs $\mu \ne 0.7813$, and $\mu =0.5432$ vs $\mu \ne 0.5432$, respectively. $^2$The null and alternative hypothesis for the variance are: $\sigma =0.00155$ vs $\sigma \ne 0.00155$, $\sigma =0.04340$ vs $\sigma \ne 0.0434$, and $\sigma =0.09900$ vs $\sigma \ne 0.099$, respectively

### Evaluation of state fidelity

We conducted a statistical analysis to assessing the reliability of the state fidelity measurement and providing insights into the variability of the fidelity of the GSA with different marked states and to examine the differences in algorithm performance across the three settings (as presented in Table 5), aiming to understand the fidelity of the population of all 3-qubit marked states.

The statistical tests on the state fidelity of a 3-qubit GSA executed on real quantum computers provide valuable insights into the fidelity of all 3-qubit marked states. The mean fidelity of 0.5432, with a standard deviation of 0.099, reflects both the average fidelity and its variability across experiments. The one-sample t-test with a 95% CI (0.4205, 0.6661) compares the observed mean fidelity against a hypothesized population mean. The resulting* p*-value of 1.00 indicates that there is insufficient evidence to reject the null hypothesis, implying that the observed mean fidelity is not significantly different from the hypothesized population mean. This suggests that the observed mean fidelity is likely representative of the population. Similarly, the hypothesis test for variance, yielding a* p*-value of 0.812, suggests no significant difference in variability between the observed sample and the hypothetical population.

This reinforces the reliability of the observed sample’s representation of the population and underscores the consistency in fidelity across different experiments. These tests provide confidence in the accuracy and reliability of the observed $\mathcal {F}_S$ values, aiding in understanding the fidelity of the population of all 3-qubit marked states.

## Quantum hardware

### Algorithm performance

**Figure 6:**
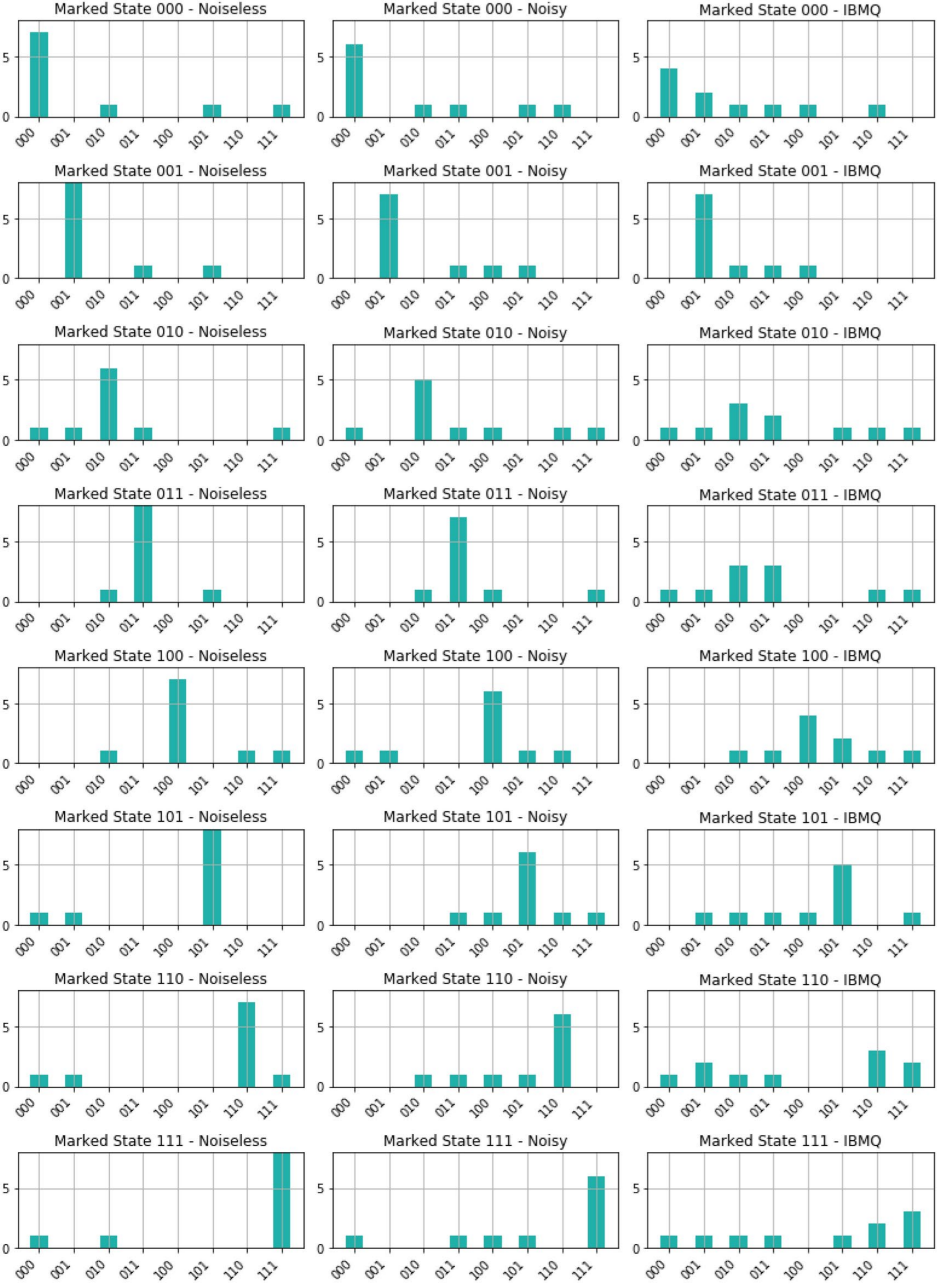
GSA performance across all 3-qubit single-marked states ($\left| 000\right\rangle , \, \left| 001\right\rangle$, $\left| 010\right\rangle , \, \left| 011\right\rangle$, $\left| 100\right\rangle , \, \left| 101\right\rangle$, $\left| 110\right\rangle , \, \text {and} \left| 111\right\rangle$) across different environments: noise-free, noisy environment, and IBM Quantum’s quantum hardware.

**Figure 7:**
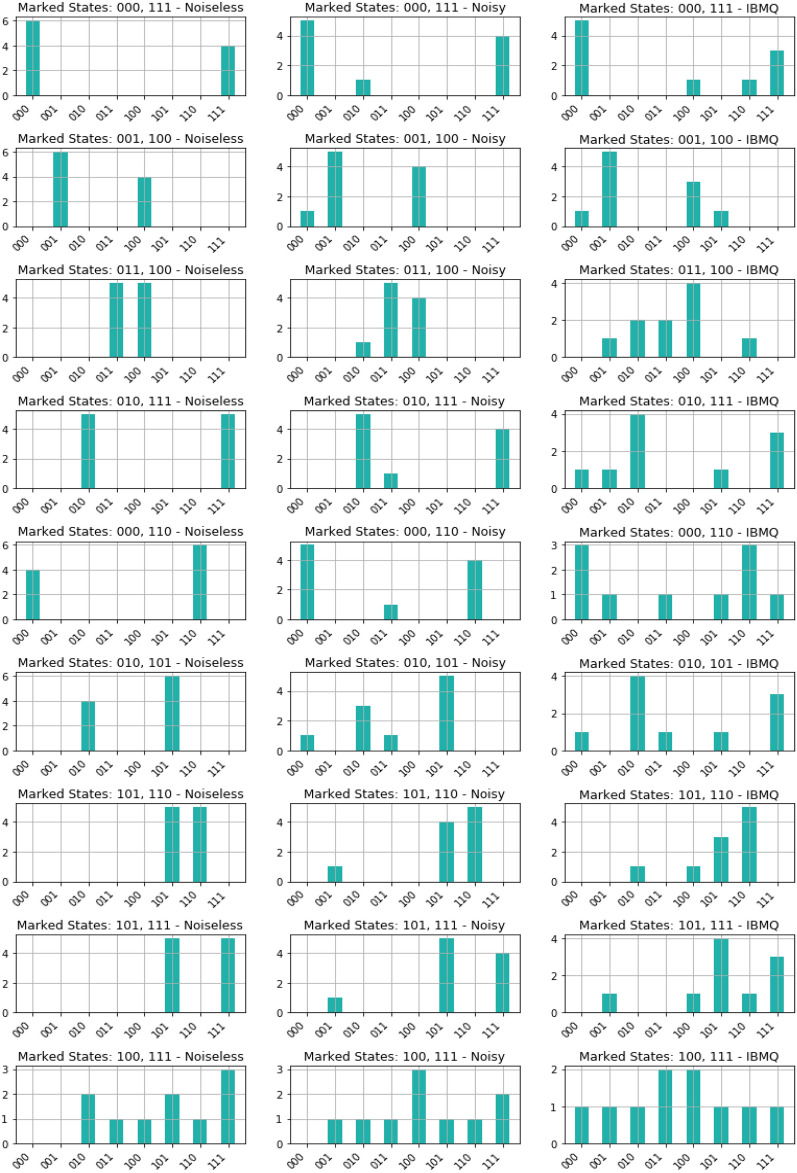
GSA performance for the nine 3-qubit two-marked states ($\left| 000\right\rangle -\, \left| 111\right\rangle$, $\left| 001\right\rangle -\, \left| 100\right\rangle$, $\left| 011\right\rangle -\, \left| 100\right\rangle$, $\left| 010\right\rangle -\, \left| 111\right\rangle$, $\left| 000\right\rangle -\, \left| 110\right\rangle$, $\left| 010\right\rangle -\, \left| 101\right\rangle$, $\left| 101\right\rangle -\, \left| 110\right\rangle$, $\left| 101\right\rangle -\, \left| 111\right\rangle$, and $\left| 100\right\rangle -\, \left| 111\right\rangle$) across different environments: noise-free, noisy environment, and IBM Quantum’s quantum hardware.

The performance of the GSA across different environments is depicted in Figure 6 and Figure 7. Figure 6 illustrates the performance of the GSA across all 3-qubit single-marked states ($\left| 000\right\rangle , \, \left| 001\right\rangle$, $\left| 010\right\rangle , \, \left| 011\right\rangle$, $\left| 100\right\rangle , \, \left| 101\right\rangle$, $\left| 110\right\rangle , \, \text {and} \left| 111\right\rangle$) across different environments: noise-free, noisy, and IBM Quantum’s quantum hardware. While Figure 7 showcases the performance for the nine 3-qubit two-marked states ( $\left| 000\right\rangle -\, \left| 111\right\rangle$, $\left| 001\right\rangle -\, \left| 100\right\rangle$, $\left| 011\right\rangle -\, \left| 100\right\rangle$, $\left| 010\right\rangle -\, \left| 111\right\rangle$, $\left| 000\right\rangle -\, \left| 110\right\rangle$, $\left| 010\right\rangle -\, \left| 101\right\rangle$, $\left| 101\right\rangle -\, \left| 110\right\rangle$, $\left| 101\right\rangle -\, \left| 111\right\rangle$, and $\left| 100\right\rangle -\, \left| 111\right\rangle$), evaluating their performance across different environments: noise-free, noisy, and real quantum hardware.

### Hardware specifications

**Figure 8:**
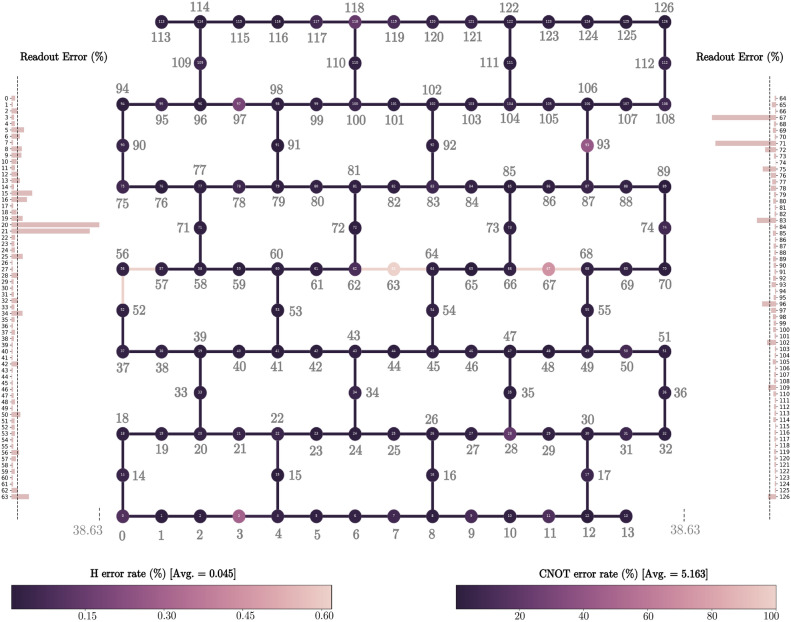
Readout error map, qubit mapping, and layout of the *ibm_sherbrooke* quantum computer. This system is built on the *Eagle r3* quantum processor, which comprises 127 superconducting transmon qubits. For detailed performance metrics, refer to Table 6.

In this section, we provide a summary of key specifications and qubit characteristics for three state-of-the-art 127-qubit superconducting quantum computers utilized in our experimental endeavors within this research. These cutting-edge quantum machines, developed by IBM Quantum, namely: *ibm_sherbrook*, *ibm_osaka*, and *ibm_kyoto*. Table 6 presents the specifications of *ibm_sherbrook*, including its model, basis gates, processor type, median ECR error, and CLOPS. The processor type is Eagle r3, and the quantum system exhibits a median ECR error of $7.565 \times 10^{-3}$, with 5000 CLOPS. While Table 7 and 8 provide a detailed summary of the essential specifications and qubit characteristics for the 127-qubit quantum computers. *ibm_osaka* and *ibm_kyoto*, respectively. Readout error map and layout of the *ibm_sherbrooke* quantum computer is shown in Figure 8.

**Table 6:** Summary of key specifications and qubit characteristics for the IBM Quantum computer with 127 qubits, named *ibm_sherbrook*. Error rates for individual gates and readout are provided, with basis gates including ECR, ID, RZ, SX, and X. The processor type is Eagle r3 (version 1.4.49). This quantum system exhibits median errors: ECR error of $7.565\times 10^{-3}$, SX error of $2.093 \times 10^{-4}$, and readout error of $1.370 \times 10^{-2}$. The median $T_1$ is 264.82 $\mu$s, and the median $T_2$ is 185.58 $\mu$s, accompanied by a performance metric of 5,000 CLOPS (CLOPS: circuit layer operations per second). Most systems support a maximum of 300 circuits and 100,000 shots, with some limitations, allowing a maximum of 100 circuits and 20,000 shots. Accessed May 18, 2024.

Properties	$T_1$ ($\mu$s)	$T_2$ ($\mu$s)	$f_{\text {qubit}}$(GHz)	$\Delta$ (GHz)	$\epsilon _{\text {readout}}$	$P_{\text {m0p1}}$	$P_{\text {m1p0}}$	$t_{\text {readout}}$ (ns)	$\epsilon _{\text {ID}}$	$\epsilon _{\text {SX}}$	$\epsilon _{\text {Pauli-X}}$
Mean	263.9271	183.6175	4.7899	-0.31039	0.02925	0.0354	0.0230	1244.44	0.00046	0.00046	0.00046
StDev	91.0896	106.6288	0.1091	0.00548	0.05786	0.0745	0.0508	0.0	0.00094	0.00094	0.00094
Min.	6.7271	6.7600	4.4552	-0.32426	0.00280	0.0034	0.0014	1244.44	0.00010	0.00010	0.00010
25%	203.1046	99.3026	4.7315	-0.31225	0.00720	0.0078	0.0051	1244.44	0.00016	0.00016	0.00016
Median	255.9965	176.4739	4.7940	-0.31093	0.01130	0.0128	0.0090	1244.44	0.00022	0.00022	0.00022
75%	336.8686	237.9368	4.8593	-0.30962	0.02465	0.0296	0.0180	1244.44	0.00032	0.00032	0.00032
Max.	466.3459	571.7688	5.0575	-0.27186	0.40130	0.4833	0.3924	1244.44	0.00618	0.00618	0.00618

Definitions and characterization of key quantum parameters in our experiments: $T_1$- the relaxation time of a qubit, measured in microseconds ($\mu s$). $T_2$- the coherence time of a qubit, measured in microseconds ($\mu s$). $f_{\text {qubit}}$- qubit frequency in (GHz). $\Delta$- qubit anharmonicity in (GHz). $\epsilon _{\text {readout}}$- readout assignment error. $P_{\text {m0p1}}$- prob meas0 prep1. $P_{\text {m1p0}}$- prob meas1 prep0. $t_{\text {readout}}$- readout length measured in (ns). $\epsilon _{\text {ID}}$- ID error. $\epsilon _{\text {SX}}$- $\sqrt{X}$ or (SX) error. $\epsilon _{\text {Pauli-X}}$- Pauli-X error

**Table 7:** Summary of key specifications and qubit characteristics for the IBM Quantum computer with 127 qubits, named *ibm_osaka*. Error rates for individual gates and readout are provided, with basis gates including ECR, ID, RZ, SX, and X. The processor type is Eagle r3 (version 1.1.7). This quantum system exhibits median errors: ECR error of $9.291 \times 10^{-3}$, SX error of $2.972 \times 10^{-4}$, and readout error of $2.320 \times 10^{-2}$. The median $T_1$ is 265.09 $\mu$s, and the median $T_2$ is 118.88 $\mu$s, accompanied by a performance metric of 5,000 CLOPS. Accessed May 27, 2024.

Properties	$T_1$ ($\mu$s)	$T_2$ ($\mu$s)	$f_{\text {qubit}}$(GHz)	$\Delta$ (GHz)	$\epsilon _{\text {readout}}$	$P_{\text {m0p1}}$	$P_{\text {m1p0}}$	$t_{\text {readout}}$ (ns)	$\epsilon _{\text {ID}}$	$\epsilon _{\text {SX}}$	$\epsilon _{\text {Pauli-X}}$
Mean	272.0908	156.9850	4.8542	-0.28497	0.0523	0.0529	0.0517	1400.00	0.00228	0.00228	0.00228
StDev	98.88077	100.2290	0.1144	0.079198	0.0784	0.0837	0.0857	0.0	0.01049	0.01049	0.01049
Min.	7.717160	5.952153	4.5680	-0.31199	0.0035	0.0040	0.0014	1400.00	0.00009	0.00009	0.00009
25%	200.5397	76.38629	4.7723	-0.30888	0.0110	0.0117	0.0101	1400.00	0.00016	0.00016	0.00016
Median	280.8633	147.2738	4.8612	-0.30749	0.0231	0.0220	0.0208	1400.00	0.00025	0.00025	0.00025
75%	342.0401	242.5020	4.9283	-0.30611	0.0591	0.0588	0.0614	1400.00	0.00043	0.00043	0.00043
Max.	469.1791	384.6830	5.1283	0.0	0.4931	0.5000	0.6896	1400.00	0.08711	0.08711	0.08711

**Table 8:** Summary of key specifications and qubit characteristics for the IBM Quantum computer with 127 qubits, named *ibm_kyoto*. Error rates for individual gates and readout are provided, with basis gates including ECR, ID, RZ, SX, and X. The processor type is Eagle r3 (version 1.2.38). This quantum system exhibits median errors: ECR error of $9.675 \times 10^{-3}$, SX error of $3.080 \times 10^{-4}$, and readout error of $1.660 \times 10^{-2}$. The median $T_1$ is 215.71 $\mu$s, and the median $T_2$ is 90.64 $\mu$s, accompanied by a performance metric of 5,000 CLOPS. Accessed May 27, 2024.

Properties	$T_1$ ($\mu$s)	$T_2$ ($\mu$s)	$f_{\text {qubit}}$(GHz)	$\Delta$ (GHz)	$\epsilon _{\text {readout}}$	$P_{\text {m0p1}}$	$P_{\text {m1p0}}$	$t_{\text {readout}}$ (ns)	$\epsilon _{\text {ID}}$	$\epsilon _{\text {SX}}$	$\epsilon _{\text {Pauli-X}}$
Mean	215.8543	117.8829	4.9666	-0.2928	0.0361	0.0375	0.0346	1400.00	0.00306	0.00306	0.00306
StDev	71.4440	85.6455	0.1301	0.0654	0.0508	0.0633	0.0462	0.0	0.02250	0.02250	0.02250
Min.	0.8683	3.6123	4.7045	-0.3120	0.0026	0.0030	0.0022	1400.00	0.00009	0.00009	0.00009
25%	173.8905	47.1228	4.8574	-0.3087	0.0096	0.0092	0.0083	1400.00	0.00019	0.00019	0.00019
Median	221.8717	94.6764	4.9596	-0.3073	0.0157	0.0154	0.0154	1400.00	0.00032	0.00032	0.00032
75%	255.0788	167.0582	5.0665	-0.3055	0.0398	0.0402	0.0377	1400.00	0.00047	0.00047	0.00047
Max.	427.4502	396.3159	5.2506	0.0	0.3153	0.4934	0.2730	1400.00	0.24625	0.24625	0.24625

It is important to emphasize that in Qiskit^68,69^, qubits are arranged in little-endian order, where the qubit with the smallest index corresponds to the least significant bit. For instance, in a three-qubit system, it is represented as $|q_2q_1q_0\rangle$. Due to this convention, in practical implementations on IBM Quantum’s hardware, the circuits displayed will appear horizontally flipped compared to their usual depiction.

## Conclusion

Quantum computers are poised at the cutting edge of technological progress, unlocking exponential computational speedups that push the boundaries of what classical computing can achieve. As quantum computing continues to evolve, Grover search algorithm stands as a pivotal advancement in the field of quantum computing, offering a revolutionary approach to solving unstructured search problems. This algorithm addresses a fundamental challenge in computing–finding a desired item in an unsorted database significantly faster than classical methods allow. Its importance cannot be overstated, as it promises exponential speedup over classical algorithms for a wide range of applications, from cryptography to database search and optimization.

Our comprehensive study sheds light on the practical implementation and performance evaluation of GSA on a state-of-the-art 127-qubits superconducting quantum computers. Through our exploration of single-solution and two-solution scenarios, we have uncovered both promising capabilities and significant challenges. Despite the presence of noise, the algorithm demonstrated a remarkable ability to maintain a high ${\mathcal {ASP}}$ and ${\mathcal {SSO}}$. For instance, in single-solution scenarios, we observed an average ${\mathcal {ASP}}$ of 78.39% and an average ${\mathcal {SSO}}$ of 82.358%, and in two-solution scenarios, an average ${\mathcal {ASP}}$ of 84.44% and an average ${\mathcal {SSO}}$ of 84.03%. However, executing the algorithm on real superconducting quantum computers revealed practical constraints, leading to decreased ${\mathcal {ASP}}$ and ${\mathcal {SSO}}$ metrics. Specifically, we noted an average ${\mathcal {ASP}}$ of 51.19% and an average ${\mathcal {SSO}}$ of 73.12% in single-solution scenarios, and an average ${\mathcal {ASP}}$ of 64.44% and an average ${\mathcal {SSO}}$ of 63.10% in two-solution scenarios.

Furthermore, our statistical analyses, which included one-sample t-tests and 95% confidence intervals, provided robust insights into the consistency and reliability of the performance metrics we observed. The one-sample t-tests comparing the observed mean ${\mathcal {ASP}}$ and ${\mathcal {SSO}}$ against hypothesized population means yielded non-significant * p*-values, indicating that the observed means of ${\mathcal {ASP}}$ and ${\mathcal {SSO}}$ are statistically representative of the population means in both scenarios. Similarly, hypothesis tests for variance produced * p*-values of 0.868 and 0.867, suggesting that there is no significant difference in the variability of ${\mathcal {ASP}}$ and ${\mathcal {SSO}}$ between our observed sample and the hypothetical population.

Additionally, we conducted five ${\mathcal {QST}}$ experiments on IBM Quantum’s 127-qubits superconducting quantum computer, *ibm_osaka*, to assess the fidelity of output states in both single-search and two-search scenarios of the GSA. Our experiments using ${\mathcal {QST}}$ provided strong confidence in the fidelity of output states. Statistical tests on the state fidelity ($\mathcal {F}_S$) of a 3-qubit GSA executed on real quantum computers revealed a mean ${\mathcal {F}}_{\mathcal {S}}$ of 0.5432. A one-sample t-test with a 95% confidence interval (0.4205, 0.6661) comparing the observed mean state fidelity against a hypothesized population mean yielded a non-significant * p*-value, indicating the observed state fidelity is likely representative of the population, with no significant variance observed, suggesting no significant difference in variability between the observed sample and the hypothetical population, reinforcing the reliability and consistency of fidelity across experiments.

Grover’s algorithm represents a monumental breakthrough in quantum computing, redefining our ability to solve unstructured search problems with unparalleled efficiency. By enabling faster discovery of a target within an unsorted database, it not only challenges the classical limits but also opens the door to transformative advancements in fields like cryptography, optimization, and large-scale data analysis. By addressing practical challenges such as noise and environmental disturbances, we can further enhance the scalability and applicability of quantum algorithms in real-world settings. Our research endeavored to contribute to the ongoing efforts to bridge the gap between theoretical developments and practical implementations, paving the way for transformative applications across various domains.

## Data Availability

The datasets generated during and/or analyzed during the current study are included within this article.
